# Enhancing stroke risk prediction in patients with transient ischemic attack: insights from a prospective cohort study implementing fast-track care

**DOI:** 10.3389/fneur.2024.1407598

**Published:** 2024-05-27

**Authors:** Valentina Barone, Matteo Foschi, Lucia Pavolucci, Francesca Rondelli, Rita Rinaldi, Marianna Nicodemo, Roberto D’Angelo, Elisabetta Favaretto, Carlotta Brusi, Benilde Cosmi, Daniela Degli Esposti, Sergio D’Addato, Stefano Bacchelli, Fabrizio Giostra, Daniela Paola Pomata, Luca Spinardi, Luca Faccioli, Gianluca Faggioli, Andrea Donti, Claudio Borghi, Pietro Cortelli, Maria Guarino, Bologna TIA Study Group

**Affiliations:** ^1^IRCCS Istituto delle Scienze Neurologiche, Bologna, Italy; ^2^Department of Biotechnological and Applied Clinical Sciences, University of L’Aquila, L’Aquila, Italy; ^3^Department of Neuroscience, S. Maria delle Croci Hospital, AUSL Romagna, Ravenna, Italy; ^4^Angiology and Blood Coagulation Unit, IRCCS Azienda Ospedaliero-Universitaria di Bologna, Bologna, Italy; ^5^Department of Cardio-Thoracic Medicine, IRCCS Azienda Ospedaliero-Universitaria di Bologna, Bologna, Italy; ^6^Department of Medical and Surgical Sciences, IRCCS Azienda Ospedaliero-Universitaria di Bologna, Bologna, Italy; ^7^Emergency Department, Medicina d’Urgenza e Pronto Soccorso, IRCCS Azienda Ospedaliero-Universitaria di Bologna, Bologna, Italy; ^8^Diagnostic and Interventional Neuroradiology Unit, IRCCS Azienda Ospedaliero-Universitaria di Bologna, Bologna, Italy; ^9^Department of Vascular Surgery, DIMEC – University of Bologna, IRCCS Azienda Ospedaliero-Universitaria di Bologna, Bologna, Italy; ^10^Pediatric Cardiology and Adult Congenital Heart Disease Program, IRCCS Azienda Ospedaliero-Universitaria di Bologna, Bologna, Italy; ^11^Department of Biomedical and Neuromotor Sciences, University of Bologna, Bologna, Italy

**Keywords:** transient ischemic attack, stroke, outcomes, fast-track, predictors, number needed to treat

## Abstract

**Background and aims:**

Fast-track care have been proved to reduce the short-term risk of stroke after transient ischemic attack (TIA). We aimed to investigate stroke risk and to characterize short- and long-term stroke predictors in a large cohort of TIA patients undergoing fast-track management.

**Methods:**

Prospective study, enrolling consecutive TIA patients admitted to a Northern Italy emergency department from August 2010 to December 2017. All patients underwent fast-track care within 24 h of admission. The primary outcome was defined as the first stroke recurrence at 90 days, 12 and 60 months after TIA. Stroke incidence with 95% confidence interval (CI) at each timepoint was calculated using Poisson regression. Predictors of stroke recurrence were evaluated with Cox regression analysis. The number needed to treat (NNT) of fast-track care in preventing 90-day stroke recurrence in respect to the estimates based on baseline ABCD_2_ score was also calculated.

**Results:**

We enrolled 1,035 patients (54.2% males). Stroke incidence was low throughout the follow-up with rates of 2.2% [95% CI 1.4–3.3%] at 90 days, 2.9% [95% CI 1.9–4.2%] at 12 months and 7.1% [95% CI 5.4–9.0%] at 60 months. Multiple TIA, speech disturbances and presence of ischemic lesion at neuroimaging predicted stroke recurrence at each timepoint. Male sex and increasing age predicted 90-day and 60-month stroke risk, respectively. Hypertension was associated with higher 12-month and 60-month stroke risk. No specific TIA etiology predicted higher stroke risk throughout the follow-up. The NNT for fast-track care in preventing 90-day stroke was 14.5 [95% CI 11.3–20.4] in the overall cohort and 6.8 [95% CI 4.6–13.5] in patients with baseline ABCD_2_ of 6 to 7.

**Conclusion:**

Our findings support the effectiveness of fast-track care in preventing both short- and long-term stroke recurrence after TIA. Particular effort should be made to identify and monitor patients with baseline predictors of higher stroke risk, which may vary according to follow-up duration.

## Background

Transient ischemic attack (TIA) represents a clinical emergency due to the increased risk of stroke and short-term cardiovascular events (1–4). Prior population-based studies showed that the risk of recurrent stroke after TIA may reach up to 17% at 3 months, with a higher incidence in the first 48 h (~50%) after the index TIA. Therefore, current guidelines recommend adopting organizational protocols based on timely diagnostic assessment and early secondary prevention, the so-called “fast-track care.” Benefits of the fast-track care were documented by several real-world studies which showed a consistent decrease in the 90-day risk of stroke recurrence after the index TIA (5, 6). However, recent evidence showed that patients with TIA may have an increased risk of recurrent cardiovascular events even beyond the first 3 months after the index event. Indeed, the 60-month incidence of recurrent stroke in patients presenting with first-ever TIA was shown to be of 9.5% (7.7% for ischemic stroke only) in the large multicentric TIA registry (2009–2011) (7), 16.1% in the Framingham cohort study (2000–2017) (8) and 6.1% in the Danish Stroke Registry (2014–2020) (9). The increased long-term stroke risk after TIA underscores the necessity of identifying predictors associated with a higher risk of long-term stroke recurrence, especially considering that factors influencing later recurrences may differ from those contributing to early ones. Hereafter, we aimed to investigate the risk of stroke and to characterize short- and long-term stroke predictors in a large population of TIA patients undergoing fast-track management.

## Methods

Study definitions are reported elsewhere (5, 10, 11). We enrolled all consecutive patients aged ≥18 years and with symptoms consistent with TIA (12) that were referred to the emergency department (ED) of the S. Orsola-Malpighi University Hospital of Bologna between August 1, 2010 and December 31, 2017. The catchment area of our ED covers a population of about 250,000 inhabitants. We included only patients with probable or definite TIA according to the National Institute of Neurological Disorders and Stroke (NINDS) criteria (13), as diagnosed after a careful examination of the medical history and neurological features. All TIA diagnoses were adjudicated by a senior vascular neurologist (MG) who decided about the study inclusion in case of discrepancy with the neurologist who initially evaluated the case.

### Fast-track work up

We implemented our fast-track diagnostic work up for all TIA patients referred to our ED starting from August 1st, 2010. This decision was informed by recent research indicating that relying solely on risk stratification using the ABCD_2_ score led to the oversight of approximately 20% of patients with significant underlying conditions posing high recurrent stroke risk (14). All patients underwent a first-line investigations within 24 h of ED admission including neurologic examination, electrocardiography (ECG), brain computed tomography (CT), blood test, blood pressure (BP) measurement, extracranial vessel study with routine color Doppler ultrasound, intracranial vessel study with transcranial color Doppler. Transthoracic echocardiography was conducted for all patients, either within 24 h for those with valvular heart disease, atrial fibrillation, recent myocardial infarction, or a history of cardiomyopathy, or scheduled as promptly as possible for other cases. ABCD_2_ score was recorded in all patients, as well as vascular risk factors and ongoing therapy on admission. Second-level investigations included prolonged ECG monitoring, CT angiography (CTA) or magnetic resonance angiography (CTA, MRA), brain MRI with diffusion-weighted (DW) sequences, transcranial Doppler with bubble study and thrombophilia screening. CTA/MRA were performed in all patients with positive Doppler ultrasound exam (i.e., detection of intracranial stenosis or symptomatic inner carotid artery stenosis, as defined by the presence of an atherosclerotic plaque determining ≥50% narrowing of the artery lumen, according to the North America Symptomatic Carotid Endarterectomy Trial [NASCET] classification) (15). Secondary prevention was initiated in all patients within 24 h of admission. The fast-track work up was driven by a senior neurovascular specialist (MG) who reviewed results of all exams and decided for optimal management.

### Follow-up and outcome adjudication

Enrolled patients were followed-up at 90 days, 12 and 60 months after the index TIA. The primary outcome was the occurrence of a first recurrent ischemic stroke at each timepoint. In patients with primary outcome the follow-up ended at the date of the recurrent stroke event, and they were considered as censored at the following timepoints. Stroke events were recorded by vascular neurologists or extracted from medical records. For patients missing scheduled on-site follow-up visits, information was retrieved by phone call or from hospital discharge records, specialty visits in the hospital, and mortality registry. Specifically, we searched for the following ICD-9- CM codes: 430 to 438 for cerebrovascular disease; 410 to 411 for acute coronary disease; 415 for pulmonary embolism; 798.1, 798.2, and 798.9 sudden death, cause unknown; and 427 for atrial fibrillation. Stroke occurrence at each follow-up was defined according to the World Health Organization (WHO) definition. The adjudication of all stroke events was confirmed by a senior vascular neurologist (MG) who reviewed clinical records and validated all outcome events.

### Statistical analysis

Categorical variables were presented as frequencies and percentages, continuous variables as mean ± standard deviation (sd) or as median (interquartile range – IQR) according to their distribution. Shapiro–Wilk and Kolmogorov–Smirnov tests were used to test the normal distribution of study variables. Comparison between categorical variables was performed using Fisher’s exact test and Pearson χ^2^ test, when appropriate. To assess the incidence of stroke at each timepoint after the index TIA and to determine confidence intervals (CIs), we employed Poisson regression analysis. Predictors of stroke at each timepoint were identified using univariate Cox regression analysis, including demographics, clinical presentation, cardiovascular risk factors, TIA etiology, neuroimaging findings, blood cholesterol and triglycerides levels. Variables were entered using a forced entry method to embed all factors which could impact on stroke risk after TIA. Subsequently, a multivariate Cox regression analysis including variables with statistical significance <0.10 was performed to identify independent stroke predictors. Results were reported as hazard ratio (HR) with 95% confidence interval (CI). The cumulative risk of stroke after TIA during the overall follow-up was estimated using Kaplan–Meier hazard function. To estimate the impact of fast-track care on the 90-day stroke risk, we calculated the incidence rate ratio (IRR) of the observed versus expected stroke incidence based on the baseline ABCD_2_ score. We also calculated the relative and absolute reduction in stroke risk compared to estimates based on the ABCD_2_ score, along with the corresponding number needed to treat (NNT) for fast-track care to prevent 90-day stroke. Lastly, a sensitivity analysis was performed to assess the risk of recurrent stroke at each timepoint in patients without ischemic lesion(s) at neuroimaging (“tissue-based” TIAs) and to evaluate the impact of fast-track care in preventing 90-day stroke in this group of patients. The statistical significance was set at a *p*-value <0.05. All analyses were performed using *R* software (version 4.2).

## Results

### Baseline characteristics

**Figure 1:**
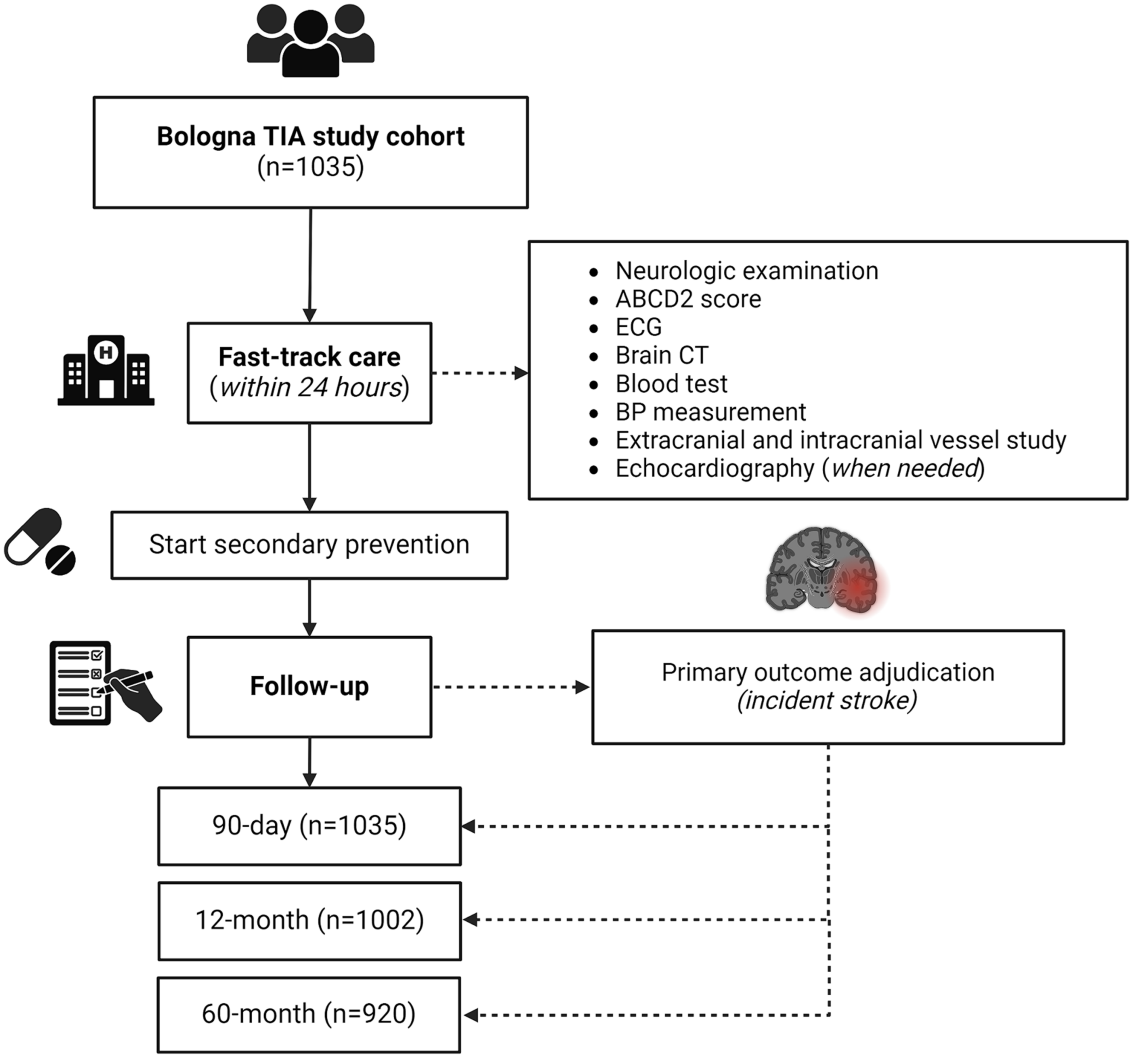
Study flow diagram. CT, computed tomography; ECG, electrocardiography; TIA, transient ischemic attack.

We included a total of 1,035 TIA patients, of whom 561 (54.2%) were male (Figure 1). For all patients, the ED admission was the first medical presentation for TIA symptoms. The mean age at TIA onset was 70.6 ± 14.5 years. The most common clinical presentation was speech disturbances (50.1%), followed by motor symptoms (49.8%). Vertebro-basilar symptoms were present in 15.4% of all TIAs. Multiple TIA accounted for 22.3% of all cases. Baseline characteristics, as well distribution of vascular risk factors and ongoing therapy on admission are summarized in Table 1.

**Table 1:** Baseline characteristics.

	Study cohort (*n* = 1,035)
*Demographic characteristics*	
Male sex, n (%)	561 (54.2)
Age – years, mean ± sd	70.6 ± 14.5
*Clinical features*	
Index TIA duration – minutes, mean ± sd	147.2 ± 276.6
Multiple TIA, n (%)	231 (22.3)
Motor symptoms, n (%)	515 (49.8)
Sensory symptoms, n (%)	372 (35.9)
Speech disturbances, n (%)	518 (50.1)
Vertebro-basilar symptoms, n (%)	159 (15.4)
Baseline ABCD_2_ score, mean ± sd	4.0 ± 1.3
High-risk TIA (ABCD_2_ score ≥ 4), n (%)	689 (66.6)
Low-risk TIA (ABCD_2_ score < 4), n (%)	346 (33.4)
Systolic blood pressure on admission – mmHg, mean ± sd	152.1 ± 26.7
Diastolic blood pressure on admission – mmHg, mean ± sd	84.7 ± 13.7
*Cardiovascular risk factors*	
Hypertension, n (%)	710 (68.6)
Diabetes mellitus, n (%)	166 (16.0)
Coronary artery disease, n (%)	36 (3.5)
Atrial fibrillation, n (%)	137 (13.2)
Cigarette smoking, n (%)	134 (12.9)
Dyslipidemia, n (%)	436 (42.1)
Prior TIA/stroke, n (%)	237 (22.9)
*Ongoing therapy on admission*	
Single antiplatelet treatment, n (%)	437 (42.2)
Dual antiplatelet treatment, n (%)	13 (12.6)
Anticoagulants, n (%)	104 (10.1)
Antihypertensive drugs, n (%)	672 (64.9)
Lipid-lowering drugs, n (%)	286 (27.6)
Antidiabetics drugs, n (%)	147 (14.2)

### Fast-track work up

Brain CT was performed in all patients on admission, while 169 patients (16.3%) underwent a second CT within 24 h of admission. Brain MRI was carried out in 180 patients (17.4%) after a median time of 3 days (IQR 2–5) of admission. All patients underwent extracranial and intracranial brain vessels study. Specifically, extracranial vessels were studied in all patients with color Doppler ultrasound, while intracranial vessels were investigated with transcranial color Doppler in 79.7% of cases. CTA or MRA were performed in 20.9% of patients. Transthoracic echocardiography was performed in 63.9% of patients. Results of the first line investigations and discharge therapies are reported in Table 2.

**Table 2:** Results of the fast-track diagnostic work up.

	Study cohort (*n* = 1,035)
*Doppler ultrasound findings*	
Extracranial inner carotid artery stenosis, n (%)	173 (16.7)
Symptomatic inner carotid artery stenosis, n (%)^a^	87 (8.4)
Intracranial stenosis, n (%)	64/815^b^ (7.9)
Patent foramen ovale, n (%)	110/200^b^ (55.0)
*Electrocardiographic findings*	
Atrial fibrillation or other cardioembolic arrhythmias, n (%)	35 (3.4%)
*Neuroimaging findings*	
Leukoaraiosis, n (%)	193 (18.7)
Presence of ischemic lesion, n (%)	48 (4.6)
*TIA etiology*^c^	
Large artery atherosclerosis, n (%)	201 (19.4)
Cardioembolic, n (%)	227 (21.9)
Small artery occlusion, n (%)	223 (21.5)
Other determined etiology, n (%)	27 (2.6)
Undetermined etiology, n (%)	357 (34.5)
*Laboratory findings*	
Total cholesterol – mg/dL, mean ± sd	197.3 ± 47.4
LDL – mg/dL, mean ± sd	122.1 ± 41.8
HDL – mg/dL, mean ± sd	53.3 ± 16.7
Triglycerides – mg/dL, mean ± sd	126.6 ± 60.2
*Secondary prevention therapy*	
Single antiplatelet treatment, n (%)	581 (56.1)
Dual antiplatelet treatment, n (%)	316 (30.5)
Anticoagulants, n (%)	176 (17.0)
Antihypertensive drugs, n (%)	824 (79.6)
Lipid-lowering drugs, n (%)	609 (58.8)
Antidiabetic drugs, n (%)	186 (18.0)
Carotid endarterectomy, n (%)	81 (7.8)
Carotid stenting, n (%)	6 (0.6)

^a^Defined as an inner carotid artery stenosis congruous with TIA symptoms ≥ 50 according to the North American Symptomatic Carotid Endarterectomy Trial (NASCET) classification.

^b^Number of patients who underwent intracranial Doppler study.

^c^TIA etiology was defined according to the Trial of ORG 10172 in Acute Stroke Treatment (TOAST) classification.

Second-level investigations were conducted in all patients with negative first-level etiological ascertainments (*n* = 357). Specifically, prolonged ECG monitoring was performed in all TIAs of undetermined etiology through 24-h (84.3%) or 48-h (15.7%) ECG Holter. Loop recorder for continuous ECG monitoring was implanted in 9 out of 357 (2.5%) patients with unknown TIA etiology. All patients with TIA of undetermined etiology underwent transthoracic echocardiography. Transcranial Doppler with bubble study and thrombophilia screening were conducted in all patients aged ≤60 years and negative first-level diagnostic work-up (83 out of 357, 23.2%).

As regards secondary prevention, in the absence of contraindications all patients with non-cardioembolic TIA started single (SAPT) or dual (DAPT) antiplatelet treatment within 24 h of admission. Specifically, SAPT was initiated in 56.1% of TIA patients, while 30.5% started DAPT. A shift toward DAPT was documented during the study period: all high-risk TIAs (ABCD_2_ ≥ 4) with non-cardioembolic etiology enrolled after 2015 were treated with short-term (21 days) DAPT, based on evidence available at the time (16). Oral anticoagulation was started in 17.0% of patients, including 35 patients (3.4%) who were diagnosed with atrial fibrillation or other cardioembolic arrhythmias at the fast-track diagnostic work up. Inner carotid artery symptomatic stenosis was detected in 87 patients (8.4%). Carotid endarterectomy or stenting were performed in 7.8 and 0.6% of TIA patients, respectively. All patients received best medical treatment for cardiovascular comorbidities. Information on secondary prevention treatment is reported in Table 2.

Among patients with low-risk TIA (346 out of 1,035, 33.4%), atrial fibrillation was detected in 8 patients (2.3%); symptomatic inner carotid artery stenosis was found in 52 patients (15.0%), while 23 patients (6.7%) had intracranial stenosis.

### Follow-up

All patients completed the 90-day follow-up, while 1,002 (96.8%) and 920 (88.9%) patients completed the 12-month and 60-month follow-up, respectively. Study flow diagram with procedures and number of patients at each follow-up is displayed in Figure 1. Overall, 131 patients died during the 60 months following the index TIA (12.6%). Death was related to stroke recurrence in 16 out of 131 cases (12.2%), while 7 out of 131 patients (5.3%) died for other vascular events (i.e., acute coronary syndrome, sudden unexpected death).

### 90-day risk of stroke and stroke predictors

The 90-day stroke incidence was 2.2% [95%CI 1.4–3.3%] (Table 3). Most stroke cases (13 out of 23, 56.5%) occurred within 48 h of the index TIA. Independent predictors of 90-day stroke recurrence included male sex (HR 2.70, [95% CI 1.06–6.91]; *p* = 0.038), multiple TIA (HR 5.85, [95% CI 2.44–14.03]; *p* < 0.001), speech disturbances (HR 2.85, [95% CI 1.16–6.98]; *p* = 0.022), and presence of ischemic lesion at neuroimaging on admission (HR 12.50, [95% CI 4.97–31.39]; *p* < 0.001). TIA etiology did not predict higher 90-day stroke risk (Supplementary Table 1).

**Table 3:** Stroke incidence after TIA.

	N of patients at each timepoint	N of incident strokes	Incidence (%) [95% CI]
48-h	1,035	13	1.3 [0.7–2.1]
90-day	1,035	23	2.2 [1.4–3.3]
12-month	1,002	29	2.9 [1.9–4.2]
60-month	920	65	7.1 [5.4–9.0]

### 12-month risk of stroke and stroke predictors

The 12-month stroke incidence was 2.9% [95% CI 1.9–4.2%] (Table 3). Independent predictors of 12-month stroke recurrence were multiple TIA (HR 3.81, [95% CI 1.85–7.81]: *p* < 0.001), speech disturbances (HR 2.31, [95% CI 1.11–4.80]; *p* = 0.026), history of hypertension (HR 3.68, [95% CI 1.37–9.88]; *p* = 0.010), and presence of ischemic lesion at neuroimaging on admission (HR 10.39, [95% CI 4.76–22.71]; *p* < 0.001). TIA etiology was not an independent predictor of 12-month stroke risk (Supplementary Table 2).

### 60-month risk of stroke and stroke predictors

At 60 months post-TIA, we found a stroke incidence of 7.1% [95% CI 5.4–9.0%] (Table 3). Increasing age (HR per 1 year increase 1.03, [95% CI 1.00–1.05]; *p* = 0.030), multiple TIA (HR 2.24, [95% CI 1.27–3.94]; *p* < 0.001), speech disturbances (HR 1.97, [95% CI 1.13–3.40]; *p* = 0.016), history of hypertension (HR 2.23, [95% CI 2.11–4.89]; *p* = 0.024), and presence of ischemic lesion at neuroimaging on admission (HR 11.29, [95% CI 5.69–22.37]; *p* < 0.001) were found to be independently associated with a higher risk of 60-month stroke recurrence. TIA etiology was not independently associated with higher 60-month stroke risk (Supplementary Table 3). The overall incidence of stroke after TIA was significantly lower in patients treated with DAPT compared to those treated with SAPT (3.5% versus 9.3%, *p* = 0.002).

The most frequent etiology of 60-month recurrent stroke (*n* = 65) was small artery occlusion (40.0%), followed by cardioembolism (24.6%), undetermined etiology (16.9%), LAA (15.4%), and other determined etiology (3.1%). We found a total concordance (100.0%) between index TIA and subsequent stroke etiologies for cardioembolism and other determined etiology. Strokes after undetermined TIAs mostly remained of unknown etiology (73.3%), but in 20.0% of patients a cardioembolic source was disclosed and a single patient (6.7%) had recurrent lacunar stroke. TIA related to small artery occlusion recurred as lacunar stroke in 91.4% of cases, while in the remaining stroke etiology was due to LAA (4.4%) or undetermined (4.4%). Lastly, LAA-related TIA usually recurred with LAA-related stroke (62.5%) but in a minority of patients the recurrent stroke event was attributable to small artery occlusion (25.0%) or cardioembolism (12.5%). Table 4 summarizes concordance between index TIA and recurrent stroke etiologies.

**Table 4:** Etiological concordance between index TIA and recurrent stroke.

	TOAST stroke (%)				
	LAA	Cardioembolic	Small artery occlusion	Other determined etiology	Undetermined etiology
TOAST index TIA					
LAA	62.5	12.5	25.0	0.0	0.0
Cardioembolic	0.0	100.0	0.0	0.0	0.0
Small artery occlusion	4.4	0.0	91.4	0.0	4.4
Other determined etiology	0.0	0.0	0.0	100.0	0.0
Undetermined etiology	0.0	20.0	6.7	0.0	73.3

LAA, large artery atherosclerosis; TOAST, Trial Of ORG 10172 in Acute Stroke Treatment.

### Kaplan–Meier hazard function of overall stroke risk after TIA

**Figure 2:**
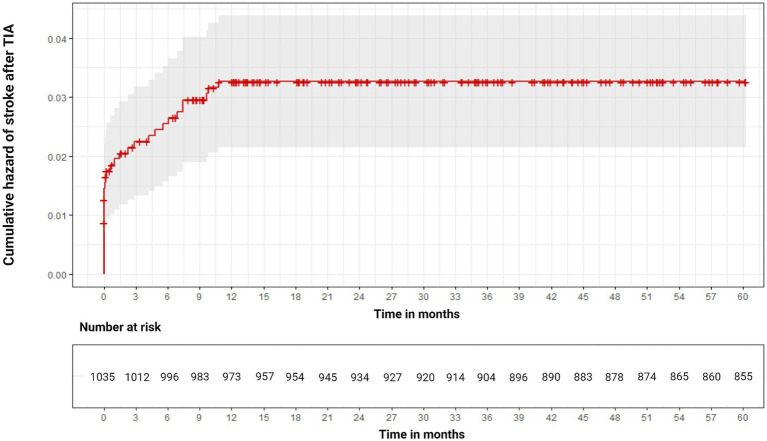
Kaplan–Meier hazard function of stroke risk after transient ischemic attack (TIA) during the overall follow-up. The upper and lower bounds of the gray area surrounding the hazard curve indicate upper and lower 95% confidence interval.

The median stroke-free survival after the index TIA was 399.4 days [95% CI 369.3–429.9]. The Kaplan–Meier hazard function confirmed that the overall risk of stroke increased over time but was particularly high during the first 90 days after TIA (35.4% of all strokes) (Figure 2).

### Impact of fast-track care in preventing 90-day stroke recurrence after TIA

The 90-day stroke risk observed within our cohort was significantly lower than based on the ABCD_2_ score (2.2% versus 9.1%, respectively; IRR: 0.2 [95% CI 0.2–0.4], *p*-value <0.001). We estimated that the fast-track care led to a reduction in the 90-day absolute and relative stroke risk of 6.9 and 75.5%, respectively. The NNT for fast-track care to prevent a 90-day stroke recurrence within the overall population was 14.5 [95% CI 11.3–20.4]. When considering only patients with baseline ABCD_2_ of 6 to 7, we calculated a reduction in the 90-day absolute and relative stroke risk of 14.6 and 82.6%, respectively, which corresponded to a NNT of 6.8 [95% CI 4.6–13.5] (Table 5).

**Table 5:** Observed versus expected 90-day stroke incidence.

ABCD_2_ score	N of patients	N of incident strokes	Observed stroke incidence [95%CI]	N of expected strokes	Expected stroke incidence [95%CI]	IRR^a^ [95%CI]	IRR^a^ *p*-value	ARR (%)	RRR (%)	NNT [95%CI]
0 to 3	346	6	1.7 [0.6–3.7]	11	3.1 [1.6–5.7]	0.5 [0.2–1.5]	0.225	1.5	45.1	69.2 [26.7–116.3]
4 to 5	559	13	2.3 [1.1–4.0]	55	9.8 [7.4–12.8]	0.2 [0.1–0.4]	<0.001	7.5	76.4	13.3 [9.7–21.1]
6 to 7	130	4	3.1 [0.8–7.7]	23	17.8 [11.2–26.5]	0.2 [0.1–0.5]	<0.001	14.6	82.6	6.8 [4.6–13.5]
Overall cohort	1,035	23	2.2 [1.4–3.3]	94	9.1 [7.34–11.1]	0.2 [0.2–0.4]	<0.001	6.9	75.5	14.5 [11.3–20.4]

ARR, absolute risk reduction; CI, confidence interval; IRR, incidence rate ratio; NNT, number needed to treat; RRR, relative risk reduction.

^a^ Observed versus expected stroke incidence.

### Sensitivity analysis

Ninety-eighty-seven patients (95.4%) without ischemic lesion(s) at neuroimaging were included in the sensitivity analysis. The recurrent stroke incidence after “tissue-based” TIAs was 1.3 [95% CI 0.7–2.3], 2.3 [95% CI 1.5–3.6], 6.2 [95% CI 4.6–8.1] at 90 days, 12 and 60 months, respectively. There were no significant differences in respect to the incidence calculated in the overall population at any follow-up (Supplementary Table 4). After excluding patients with positive neuroimaging, we calculated a NNT for the fast-track care in preventing 90-day stroke of 12.8 [95% CI 11.5–13.3] within the overall population (absolute risk reduction 7.4%, relative risk reduction 85.6%).

## Discussion

Our prospective study demonstrated an increasing incidence of stroke over time (from 2.2% at 90 days to 7.1% at 60 months) in a large population of TIA patients undergoing fast-track care. Predictors of post-TIA stroke recurrence were different between the short- and long-term follow-up. Specifically, we found that multiple TIA, speech disturbances at TIA onset and presence of ischemic lesion at neuroimaging performed on ED admission were independently associated with an increased risk of stroke at each timepoint. On the other hand, male sex and increasing age were independent predictors of stroke occurrence at 90-day and 60-month, respectively, while history of hypertension independently predicted both the 90-day and 60-month stroke risk. Lastly, the overall incidence of stroke at 90 days within our cohort was lower than expected based on the baseline ABCD_2_ score. We estimated NNT for fast-track care in preventing stroke of 14.5 [95% CI 11.3–20.4] in the overall cohort and of 6.8 [95% CI 4.6–13.5] in patients with baseline ABCD_2_ of 6 to 7.

Considering baseline characteristics, the mean age of TIA patients within our cohort (70.6 years) was higher compared to the prior large multicentric TIA registry (66.1 years) (6), but in line with a recent European nationwide study which found a mean age at TIA onset of 70.8 years (9). The relatively advanced mean age of our population, coupled with our comprehensive investigation of TIA causes through fast-track care, could explain the peculiar distribution of TIA etiologies observed within our cohort. Specifically, we noted a higher proportion of patients experiencing cardioembolic TIAs (21.9%) compared to prior population-based studies (17, 18). Conversely, while the prevalence of TIA cases with undetermined etiology (34.5%) was comparatively lower in respect to prior studies (19, 20), it still remained consistently high. This highlights the persistent challenge of identifying a well-defined cause in a significant percentage of TIA patients, despite implementing a thorough diagnostic work-up.

The short-term risk of stroke observed within our cohort (2.2%) was one of the lowest if compared to current literature, especially when considering studies predating the implementation of fast-track care for patients with TIA (21–25). A systematic review conducted by Valls et al. in 2016 analyzed several studies applying accelerated TIA management from 2007 to 2015 and reported a pooled 90-day stroke risk of 3.4% (26). Furthermore, studies conducted prior to the implementation of fast-track care (2000–2003) estimated a 90-day stroke risk ranging from 12 to 20% (27, 28). Although the cumulative risk of stroke increased over time, we found a lower incidence of stroke events at each timepoint in respect to prior cohort studies. Specifically, our 12-month stroke incidence (3.3%) was lower than those observed in the Aarhus study (25) and in the large multicentric TIA registry (6) (4.2, and 5.1%, respectively). Similarly, the observed 60-month stroke rate observed within our cohort (7.1%) was lower than those reported in the more recent update of the same TIA registry (9.5%) (7). Importantly, our sensitivity analysis revealed no significant differences in recurrent stroke incidence following TIA at each timepoint when excluding patients with ischemic lesion(s) on neuroimaging. This suggests a comparable stroke risk within our population whether employing a “time-based” or “tissue-based” definition of the index TIA. This finding aligns with recent observations from a prospective cohort study showing substantial stroke risk even after tissue-negative TIAs (29).

Our recorded stroke risk, along with the observation that 56.5% of all 90-day strokes occurred within the first 48 h after TIA, corroborate the need of immediate care to facilitate early diagnosis and initiate effective secondary prevention. This effort may have the potential not only to reduce early stroke risk but also to ameliorate long-term TIA prognosis. Accordingly, it is crucial to extend the follow-up monitoring for patients who have experienced a TIA even beyond the initial high-risk period.

Predictors of stroke incidence after TIA have been scarcely characterized especially in the long-term. In line with prior prospective studies (10, 11), our analysis confirmed that multiple TIA, speech disturbances and presence of ischemic lesion at neuroimaging show the strongest association with an increased risk of stroke independently of the follow-up duration. The association between multiple TIA presentation and increased stroke risk is well-established, especially for patients with recurrent lacunar syndrome (11). Similarly, TIA with lesion has emerged as a condition of very high risk (10). This recognition might carry significant implications for selecting the most suitable antiplatelet regimen to prevent early recurrent stroke, since dual antiplatelet therapy might offer enhanced efficacy in this specific TIA subtype (30). In terms of speech disturbances, the variable localization of this non-specific symptom might delay targeted secondary prevention, thus increasing the subsequent stroke risk, as noted elsewhere (31). Additionally, concerning demographic factors, male sex and increasing age independently predicted higher 90-day and 60-month stroke risk, respectively. This finding aligns with observations from prior studies, which found a significant interaction between sex and age in determining post-TIA stroke risk (32, 33). In particular, whilst male sex may be related with a higher burden or greater severity of cardiovascular risk factors which concur in enhancing the short-term risk of stroke, sex differences might attenuate in the long-term, when increasing age overcomes biological disparities in stroke pathophysiology. Remarkably, when considering the 12-month and 60-month follow-up, history of hypertension emerged as an independent risk factor for stroke recurrence. This association was also observed in another prospective study with long-term follow-up (31). It is likely that the chronic injury inflicted by hypertension on brain vessels may, to some extent, resist secondary preventive interventions, which may effectively mitigate the short-term risk of stroke but could prove insufficient in preventing late recurrences.

Of interest, no specific TIA etiology was found to independently predict a higher stroke risk within our population. Prior studies found that large artery atherosclerosis (LAA) TIA etiology was independently associated with a higher short-term risk of stroke recurrence, (34, 35) while no etiological TIA subtype has been identified as robust predictor of increased stroke risk in the long-term (36). We may speculate that implementing urgent measures to prevent ischemic recurrences within our cohort, as provided by our fast-track care, may have mitigated the unfavorable effect of LAA etiology on future stroke risk. Additionally, as a shift toward DAPT was documented within our cohort during the study period, we cannot exclude that the impact of TIA etiology on stroke risk may have been nuanced by the rising utilization of DAPT over time. Indeed, we observed a significantly lower risk of recurrent stroke events in patients treated with DAPT versus SAPT throughout the study follow-up. This finding underscores the critical importance of strict adherence to current international guidelines recommending early DAPT as the best option for preventing ischemic recurrences in patients with non-cardioembolic high-risk (ABCD_2_ ≥ 4) TIA (37). Furthermore, within this evolving treatment paradigm, expedited diagnostic protocols can offer crucial insights for treatment planning. Swift identification of particular TIA etiologies (i.e., cardioembolism or symptomatic LAA) can significantly refine patient selection for prompt interventions like DAPT, anticoagulation, or other tailored approaches, regardless of their initial ABCD_2_ score. In fact, within our cohort, a relevant proportion of patients with baseline ABCD_2_ score < 4 was diagnosed with underlying high-risk conditions such as atrial fibrillation (2.3%), symptomatic inner carotid artery stenosis (15.0%), or intracranial stenosis (6.7%).

Lastly, we estimated that within our cohort the fast-track care was highly effective in reducing the expected risk of recurrent stroke based on the baseline ABCD_2_ score. In particular, the NNT for fast-track care in preventing future stroke was 14.5 [95% CI 11.3–20.4] in the overall cohort and 6.8 [95% CI 4.6–13.5] in the high-risk TIA subgroup (ABCD_2_ of 6 to 7). The sensitivity analysis excluding patients with ischemic lesion(s) at neuroimaging showed even lower NNT in the overall cohort 12.8 [95% CI 11.5–13.3] for “tissue-based” TIAs. Our estimated NNT for fast-track care indicates a potentially greater magnitude of benefit compared to hospitalization in an acute stroke unit, where the estimated NNT in preventing bad outcomes typically ranges between 10 to 25 (38). This result strengthens the value of fast neurovascular specialist-driven ED management in improving outcomes of TIA patients, as previously also observed in an outpatient clinic setting (39).

Strengths of our study lie in its prospective design, the large cohort dimension, and the comprehensive evaluation of enrolled patients, as ensured by the systematic application of the fast-track diagnostic work up, which was implemented at our site, well before it was recommended by the National Institute for Health and Care Excellence (NICE) guidelines (2019) (40). However, there are some limitations that need to be acknowledged. Firstly, due to the lack of systematic data collection on TIA patients before the implementation of fast-track care at our site (2010), we were unable to rely on a control group of TIA patients to directly compare figures on fast-track care effectiveness. As a consequence, we could not assess the extent of observed benefit on stroke risk following TIA in relation to the timeliness or thoroughness of the work-up. Secondly, on the one side, the small number of stroke recurrences in the short-term follow-up might reflect the effectiveness of fast-track care in reducing early stroke risk after TIA, but on the other it might limit the reliability of the identified 90-day stroke predictors. Additionally, we did not record information on patients’ adherence to prescribed medications. Therefore, identified predictors of stroke recurrence should be interpreted in light of this limitation. It is also important to note that advanced and more sensitive neuroimaging (i.e., Diffusion-Weighted MRI) was precluded to most patients of our cohort (>80%). Consequently, the number of patients with ischemic lesion(s) at neuroimaging may have been consistently underestimated limiting the reliability of our sensitivity analysis on “tissue-based” TIAs. However, the adoption of a “time-based” rather than a “tissue-based” definition for TIA diagnosis ensures comparability of our findings with prior prospective studies evaluating stroke predictors. Moreover, in our fast-track workup, the majority of patients underwent color Doppler ultrasound to assess both extra- and intracranial vessels, while CTA/MRA were predominantly reserved for those with significant brain vessel stenosis. This strategy leverages the rapid availability of Doppler exams within 24 h at our facility. However, it may not be suitable for different organizational settings where CTA, contextual with admission non-contrast CT, could offer a quicker and more accessible evaluation, as highlighted in a recent scientific statement by the American Heart Association (41). Lastly, we cannot exclude that the incidence of recurrent strokes recorded in our study has been underestimated because we did not search all regional hospitals but only our hospital records.

In conclusion, our findings provide compelling evidence for the efficacy of fast-track care in mitigating both the short- and long-term risk of stroke recurrence following a TIA. Particular effort should be made to identify and monitor patients with baseline predictors of higher stroke risk, which may vary according to the follow-up duration. This underscores the necessity for tailored risk assessment and intervention strategies to prevent the occurrence of future ischemic events after TIA, even beyond the initial high-risk period.

## Data availability statement

The raw data supporting the conclusions of this article are available at the URL: https://zenodo.org/records/11096699.

## Ethics statement

The studies involving humans were approved by S. Orsola-Malpighi University Hospital Research Ethics Board. The studies were conducted in accordance with the local legislation and institutional requirements. The participants provided their written informed consent to participate in this study.

## Author contributions

VB: Data curation, Writing – original draft, Writing – review & editing. MF: Data curation, Formal analysis, Methodology, Visualization, Writing – original draft, Writing – review & editing. LP: Writing – original draft, Writing – review & editing. FR: Writing – original draft, Writing – review & editing. RR: Writing – original draft, Writing – review & editing. MN: Writing – original draft, Writing – review & editing. RD’A: Writing – original draft, Writing – review & editing. EF: Writing – review & editing, Writing – original draft. CBr: Writing – review & editing, Writing – original draft. BC: Writing – review & editing, Writing – original draft. DD: Writing – review & editing, Writing – original draft. SD’A: Writing – original draft, Writing – review & editing. SB: Writing – original draft, Writing – review & editing. FG: Writing – review & editing, Writing – original draft. DP: Writing – review & editing, Writing – original draft. LS: Writing – original draft, Writing – review & editing. LF: Writing – original draft, Writing – review & editing. GF: Writing – original draft, Writing – review & editing. AD: Writing – review & editing, Writing – original draft. CBo: Writing – review & editing, Writing – original draft. PC: Writing – original draft, Writing – review & editing. MG: Conceptualization, Supervision, Validation, Writing – original draft, Writing – review & editing.
