# Supplementary material for: Enhancing stroke risk prediction in patients with transient ischemic attack: insights from a prospective cohort study implementing fast-track care
Source: Front Neurol. 2024 May 27;15:1407598. doi: 10.3389/fneur.2024.1407598 (PMC11163114; doi:10.3389/fneur.2024.1407598)
Supplement: Supplementary file 1 [file Table_1.docx]

**Supplementary Material**

**Supplementary Table 1. Univariate and multivariate Cox regression analysis of 90-day stroke predictors.**

|  | **Univariate analysis** | | **Multivariate analysis** | |
| --- | --- | --- | --- | --- |
| **Variable** | **HR [95% CI]** | **P value** | **HR [95% CI]** | **P value** |
| Male sex | 2.58 [1.02-6.49] | **0.032** | 2.70 [1.06-6.91] | **0.038** |
| Age – per 1 year increase | 1.00 [0.97-10.3] | 0.947 |  |  |
| Index TIA duration – per 1 minute increase | 1.00 [0.99-1.00] | 0.109 |  |  |
| Multiple TIA | 2.27 [4.25-22.21] | **<0.001** | 5.85 [2.44-14.03] | **<0.001** |
| Motor symptoms | 3.05 [1.21-7.68] | **0.011** | 1.59 [0.59-4.03] | 0.360 |
| Sensory symptoms | 1.04 [0.45-2.37] | 0.928 |  |  |
| Speech disturbances | 2.52 [1.05-6.09] | **0.030** | 2.85 [1.16-6.98] | **0.022** |
| Vertebro-basilar symptoms | 0.49 [0.12-2.08] | 0.333 |  |  |
| Systolic blood pressure on admission – per 1 mmHg increase | 1.01 [0.99-1.02] | 0.193 |  |  |
| Diastolic blood pressure on admission – per 1 mmHg increase | 1.02 [0.99-1.05] | 0.101 |  |  |
| Hypertension | 1.71 [0.63-4.56] | 0.289 |  |  |
| Diabetes mellitus | 1.09 [0.37-3.12] | 0.872 |  |  |
| Coronary artery disease | 0.46 [0.11-1.94] | 0.236 |  |  |
| Atrial fibrillation | 0.55 [0.13-2.35] | 0.382 |  |  |
| Cigarette smoking | 2.21 [0.79-2.21] | 0.119 |  |  |
| Dyslipidemia | 0.78 [0.34-1.78] | 0.547 |  |  |
| Prior TIA/stroke | 0.83 [0.30-2.22] | 0.703 |  |  |
| TIA etiology: large artery atherosclerosis | 1.00 (ref.) | - |  |  |
| TIA etiology: cardioembolic | 0.29 [0.06-1.44] | 0.130 |  |  |
| TIA etiology: small artery occlusion | 1.67 [0.62-4.53] | 0.308 |  |  |
| TIA etiology: other determined etiology | 1.39 [0.17-11.58] | 0.760 |  |  |
| TIA etiology: undetermined etiology | 0.41 [0.12-1.44] | 0.164 |  |  |
| Extracranial inner carotid artery stenosis | 1.91 [0.79-4.60] | 0.172 |  |  |
| Leukoaraiosis | 3.32 [1.47-7.48] | **0.037** | 2.00 [0.85-4.69] | 0.112 |
| Presence of ischemic lesion | 23.20 [10.19-52.79] | **<0.001** | 12.50 [4.97-31.39] | **<0.001** |
| Total cholesterol – per 1 mg/dL increase | 1.00 [0.99-1.01] | 0.965 |  |  |
| LDL – per 1 mg/dL increase | 1.00 [0.99-1.01] | 0.832 |  |  |
| HDL – per 1 mg/dL increase | 0.99 [0.97-1.02] | 0.667 |  |  |
| Triglycerides – per 1 mg/dL increase | 1.00 [0.99-1.01] | 0.937 |  |  |

CI: confidence interval; HR: hazard ratio.

**Supplementary Table 2. Univariate and multivariate Cox regression analysis of 12-month stroke predictors.**

|  | **Univariate analysis** | | **Multivariate analysis** | |
| --- | --- | --- | --- | --- |
| **Variable** | **HR [95% CI]** | **P value** | **HR [95% CI]** | **P value** |
| Male sex | 1.50 [0.74-3.05] | 0.253 |  |  |
| Age – per 1 year increase | 1.01 [0.98-1.03] | 0.530 |  |  |
| Index TIA duration – per 1 minute increase | 1.00 [0.99-1.01] | 0.886 |  |  |
| Multiple TIA | 5.41 [2.73-10.71] | **<0.001** | 2.70 [1.25-5.88] | **0.013** |
| Motor symptoms | 2.75 [1.27-5.91] | **0.006** | 2.07 [0.93-4.60] | 0.073 |
| Sensory symptoms | 0.88 [0.42-1.81] | 0.726 |  |  |
| Speech disturbances | 2.02 [0.98-4.16] | **0.050** | 2.40 [1.14-5.07] | **0.022** |
| Vertebro-basilar symptoms | 0.76 [0.27-2.15] | 0.585 |  |  |
| Systolic blood pressure on admission – per 1 mmHg increase | 1.00 [0.99-1.01] | 0.645 |  |  |
| Diastolic blood pressure on admission – per 1 mmHg increase | 1.00 [0.98-1.03] | 0.840 |  |  |
| Hypertension | 2.57 [0.99-6.65] | **0.003** | 3.77 [1.34-10.66] | **0.012** |
| Diabetes mellitus | 1.69 [0.76-3.74] | 0.220 |  |  |
| Coronary artery disease | 1.56 [0.11-1.94] | 0.292 |  |  |
| Atrial fibrillation | 0.63 [0.19-2.06] | 0.412 |  |  |
| Cigarette smoking | 1.84 [0.79-4.23] | 0.153 |  |  |
| Dyslipidemia | 1.28 [0.64-2.52] | 0.487 |  |  |
| Prior TIA/stroke | 1.06 [0.48-2.35] | 0.887 |  |  |
| TIA etiology: large artery atherosclerosis | 1.00 (ref.) |  |  |  |
| TIA etiology: cardioembolic | 0.29 [0.08-1.07] | **0.064** | 0.34 [0.09-1.30] | 0.115 |
| TIA etiology: small artery occlusion | 1.54 [0.67-3.51] | 0.309 | 1.32 [0.56-3.12] | 0.522 |
| TIA etiology: other determined etiology | 1.65 [0.36-7.64] | 0.522 | 1.72 [0.32-9.14 | 0.522 |
| TIA etiology: undetermined etiology | 0.25 [0.08-0.80] | **0.020** | 0.42 [0.12-1.41] | 0.159 |
| Extracranial inner carotid artery stenosis | 1.57 [0.71-3.48] | 0.287 |  |  |
| Leukoaraiosis | 1.99 [0.95-4.18] | **0.085** | 1.41 [0.66-3.02] | 0.368 |
| Presence of ischemic lesion | 12.03 [5.83-24.83] | **<0.001** | 9.40 [4.09-21.58] | **<0.001** |
| Total cholesterol – per 1 mg/dL increase | 0.99 [0.98-1.01] | 0.223 |  |  |
| LDL – per 1 mg/dL increase | 1.00 [0.99-1.01] | 0.958 |  |  |
| HDL – per 1 mg/dL increase | 1.01 [0.97-1.02] | 0.988 |  |  |
| Triglycerides – per 1 mg/dL increase | 1.00 [0.99-1.01] | 0.833 |  |  |

CI: confidence interval; HR: hazard ratio.

**Supplementary Table 3. Univariate and multivariate Cox regression analysis of 60-month stroke predictors.**

|  | **Univariate analysis** | | **Multivariate analysis** | |
| --- | --- | --- | --- | --- |
| **Variable** | **HR [95% CI]** | **P value** | **HR [95% CI]** | **P value** |
| Male sex | 1.21 [0.72-2.02] | 0.468 |  |  |
| Age – per 1 year increase | 1.03 [1.01-1.05] | **0.009** | 1.03 [1.00-1.05] | **0.039** |
| Index TIA duration – per 1 minute increase | 1.00 [0.99-1.01] | 0.262 |  |  |
| Multiple TIA | 2.69 [1.56-4.64] | **<0.001** | 2.12 [1.20-3.74] | **0.010** |
| Motor symptoms | 2.06 [1.56-4.64] | **0.007** | 1.71 [0.94-2.95] | 0.063 |
| Sensory symptoms | 0.71 [0.41-1.24] | 0.223 |  |  |
| Speech disturbances | 2.11 [1.23-3.61] | **0.004** | 1.93 [1.13-3.27] | **0.015** |
| Vertebro-basilar symptoms | 0.71 [0.32-1.56] | 0.368 |  |  |
| Systolic blood pressure on admission – per 1 mmHg increase | 1.00 [0.99-1.02] | 0.450 |  |  |
| Diastolic blood pressure on admission – per 1 mmHg increase | 1.00 [0.99-1.02] | 0.437 |  |  |
| Hypertension | 2.02 [1.05-3.88] | **0.024** | 2.55 [1.24-5.12] | **0.011** |
| Diabetes mellitus | 1.56 [0.84-2.89] | 0.174 |  |  |
| Coronary artery disease | 1.52 [0.84-2.78] | 0.182 |  |  |
| Atrial fibrillation | 0.42 [0.15-1.14] | 0.533 |  |  |
| Cigarette smoking | 1.31 [0.66-2.57] | 0.456 |  |  |
| Dyslipidemia | 1.00 [0.60-1.66] | 0.993 |  |  |
| Prior TIA/stroke | 1.09 [0.61-1.93] | 0.779 |  |  |
| Extracranial inner carotid artery stenosis | 1.18 [0.63-2.18] | 0.608 |  |  |
| TIA etiology: large artery atherosclerosis | 1.00 (ref.) | - | 1.00 (ref.) | - |
| TIA etiology: cardioembolic | 0.48 [0.21-1.10] | **0.084** | 0.80 [0.24-1.25] | 0.150 |
| TIA etiology: small artery occlusion | 1.31 [0.69-2.47] | 0.412 | 0.43 [0.66-2.44] | 0.482 |
| TIA etiology: other determined etiology | 0.91 [0.21-3.97] | 0.903 | 0.81 [0.30-6.99] | 0.652 |
| TIA etiology: undetermined etiology | 0.51 [0.25-1.03] | **0.061** | 0.38 [0.45-1.98] | 0.883 |
| Leukoaraiosis | 2.49 [1.45-4.26] | **0.002** | 1.60 [0.93-2.76] | 0.087 |
| Presence of ischemic lesion | 8.58 [4.70-15.66] | **<0.001** | 10.96 [5.44-22.07] | **<0.001** |
| Total cholesterol – per 1 mg/dL increase | 1.00 [0.99-1.01] | 0.204 |  |  |
| LDL – per 1 mg/dL increase | 1.00 [0.99-1.01] | 0.635 |  |  |
| HDL – per 1 mg/dL increase | 0.99 [0.98-1.01] | 0.553 |  |  |
| Triglycerides – per 1 mg/dL increase | 1.00 [0.99-1.01] | 0.656 |  |  |

CI: confidence interval; HR: hazard ratio.

**Supplementary Table 4. Sensitivity analysis: stroke incidence after “tissue-based” TIAs**

|  | **N of patients at each timepoint** | **N of incident strokes** | **Incidence (%)**  **[95% CI]** | **IRR^a^ [95% CI]** | **P value** |
| --- | --- | --- | --- | --- | --- |
| 48-h | 987 | 8 | 0.8 [0.4-1.6] | 0.7 [0.3-1.6] | 0.326 |
| 90-day | 987 | 13 | 1.3 [0.7-2.3] | 0.6 [0.3-1.2] | 0.127 |
| 12-month | 938 | 22 | 2.3 [1.5-3.6] | 0.8 [0.5-1.4] | 0.456 |
| 60-month | 823 | 51 | 6.2 [4.6-8.1] | 0.9 [0.6-1.3] | 0.483 |

CI: confidence interval; IRR: incidence rate ratio. a: “tissue-based” TIAs versus overall TIAs.
